# *Murbeckiella* is dead, long live *Oreophyton*: origin and systematics of tribe Oreophytoneae (Brassicaceae)

**DOI:** 10.1093/aob/mcaf115

**Published:** 2025-06-11

**Authors:** Nora Walden, Guilhem Mansion, Hektor Schnorf, Marcus A Koch, Alessia Guggisberg

**Affiliations:** Centre for Organismal Studies, Heidelberg University, D-69120 Heidelberg, Germany; Institute of Integrative Biology, ETH Zurich, CH-8092 Zurich, Switzerland; Institute of Integrative Biology, ETH Zurich, CH-8092 Zurich, Switzerland; Centre for Organismal Studies, Heidelberg University, D-69120 Heidelberg, Germany; Institute of Integrative Biology, ETH Zurich, CH-8092 Zurich, Switzerland

**Keywords:** Biogeography, *Murbeckiella*, nomenclature, *Oreophyton*, Oreophytoneae, Paralog PhyloGenomics, phylogenomics, plastome, systematics, taxonomy

## Abstract

**Background and Aims:**

The small genus *Murbeckiella* from the Brassicaceae family underwent manifold taxonomic changes reflecting morphological uncertainties, but also unusual biogeographical distribution patterns ranging from Spain and northern Africa to Caucasus. Close relationships with *Oreophyton* have been proposed, and both genera have been placed in the orphan tribe Oreophytoneae. We explore phylogenetic relationships to unravel and revise present-day taxonomic treatments, propose a biogeographical framework and contribute to our understanding of the local endemic *Murbeckiella omissa* from France.

**Methods:**

To infer general phylogenetic patterns, short-read whole-genome sequencing (WGS) data were mapped to a 1000 nuclear gene target dataset. In addition, divergence time estimates were inferred from plastome coding sequences. Finally, the recently introduced Paralog PhyloGenomics (PPG) approach was tested to detect putative hybrids and resolve reticulate evolutionary patterns at the polyploid level.

**Key Results:**

The PPG concept resolved phylogenetic relationships among diploids and polyploids successfully. *Murbeckiella* appeared poly- and paraphyletic and the majority of species have been taxonomically integrated into a newly defined genus, *Oreophyton*. One single species, *Murbeckiella sousae*, is placed in a clade of a distinct tribe, Arabideae, and is awaiting future taxonomic revision. Biogeographical evidence suggests an origin of the tribe in Anatolia or adjacent Levant with crown group diversification of the different species starting in the early Pleistocene.

**Conclusions:**

The genus *Murbeckiella* is a suitable system to apply WGS methods to unravel reticulate evolutionary histories of young species complexes. Its small genome allows deep sequencing at high quality, which may facilitate future studies illustrating the genomic footprints and genetic backgrounds of range expansion and transcontinental migration as well as adaptation in rapidly changing alpine environments.

## INTRODUCTION

With rising temperatures due to global warming, plant species have shifted their ranges to higher latitudes and altitudes (Grabheer *et al*., 1994; Lenoir *et al*., 2008; Steinbauer *et al*., 2018), with potentially unprecedented consequences for community interactions (Tylianakis *et al*., 2008), species integrity (Gómez *et al*., 2015) or population growth rates (Cotto *et al*., 2017). The situation is particularly exacerbated in mountain areas, because orophytes will experience the strongest environmental changes (Engler *et al*., 2011; Dullinger *et al*., 2012). Orogenic belts that today experience a concomitant decrease in precipitation, such as the Spanish Pyrenees and Moroccan Atlas Mountains, are predicted to undergo drastic floristic changes (Engler *et al*., 2011).

**Fig. 1.**
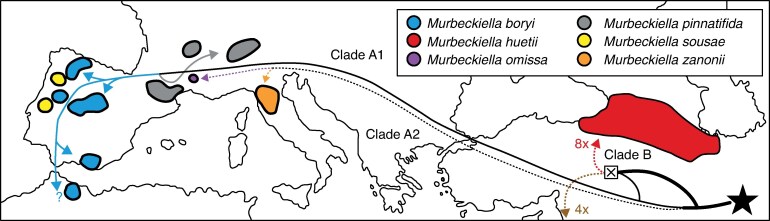
Map representing geographical distribution and tentative biogeographical scenario for taxa belonging to tribe Oreophytoneae (redrawn from Rothmaler, 1939). The star represents the ancestor of tribe Oreophytoneae; the cross represents hybridisation between the ancestral ‘ghost’ lineage and (*Murbeckiella pinnatifida*/*M. boryi*) subclade A1 at the origin of the octoploid *M. huetii* (8x) and tetraploid *Oreophyton falcatum* (4x; distribution not shown).

From an evolutionary viewpoint, high mountain systems are reminiscent of island-like regions that are generally colonized via long-distance dispersals and/or rapid range expansions (Carlquist, 1974). As a result of increased geographical isolation, limited dispersal opportunities and environmental harshness, cold-adapted plants often exhibit reduced genetic diversity, slow growth rates and long life cycles, making them even more vulnerable to anthropogenic disturbances (Reed and Frankham, 2003; Waters *et al*., 2013; Cotto *et al*., 2017; Love *et al*., 2023). In this context, *Murbeckiella* (Brassicaceae) appears to be an ideal model for the study of fragile alpine ecosystems (Woolbright *et al*., 2014; Love *et al*., 2023). Indeed, with six species currently distributed in the alpine zones of the siliceous/volcanic mountain ranges of southern/central Europe, northwestern Africa and southwestern Asia (Fig. 1; Schulz, 1924, 1936; Rothmaler, 1939), the genus seems particularly suitable for inferring the evolution of orophytes in the context of climate change and habitat reduction.

The most widespread species *Murbeckiella pinnatifida* (Lam.) Rothm. is an alpine diploid (2*n* = 2*x* = 16) that occurs in the southwestern Alps, the central Massif Central (Mont-Dore, Mounts of Cantal) and the Pyrenees; potential outposts in Transylvania have not been confirmed so far (Rouy and Foucaud, 1895; Rothmaler, 1939). The diploid *Murbeckiella boryi* (Boiss.) Rothm. (2*n* = 2*x* = 16) morphologically resembles *M. pinnatifida* and occurs in the Iberian Peninsula (Cantabrian Mountains, Iberian and Central System, Sierra Nevada) and the Moroccan Rif. *Murbeckiella huetii* (Boiss.) Rothm. is an octoploid species (2*n* = 8*x* = 64; M. A. Lysák, Central European Institute of Technology (CEITEC), Masaryk University, Czech Republic, pers. comm.) that can be encountered in the Caucasus and eastern Anatolia. Finally, the remaining three species show very small ranges and unclear ploidy levels. *Murbeckiella sousae* Rothm. is endemic to the northern (Serra da Freita and Serra do Marão) and central (Serra da Lousã) mountains of Portugal; *M. zanonii* (Ball) Rothm. can be encountered in the northern (Tuscan–Emilian) Apennines, whereas *M. omissa* B.P.R.Chéron is a recently described taxon occurring in the eastern Massif Central (Cévennes; Chéron, 2020). Populations of this putative diploid species (2*n* = 2*x* = c. 16; B. P. R. Chéron, France, pers. comm.) have long been confused with *M. zanonii* and its taxonomic status needs to be evaluated. This taxon was not included in BrassiBase (Koch *et al*., 2012; Kiefer *et al*., 2014), but an updated species checklist including *M. omissa* was recently integrated into the *World Flora Online* release of December 2024 (WFO, 2025).

**Fig. 2.**
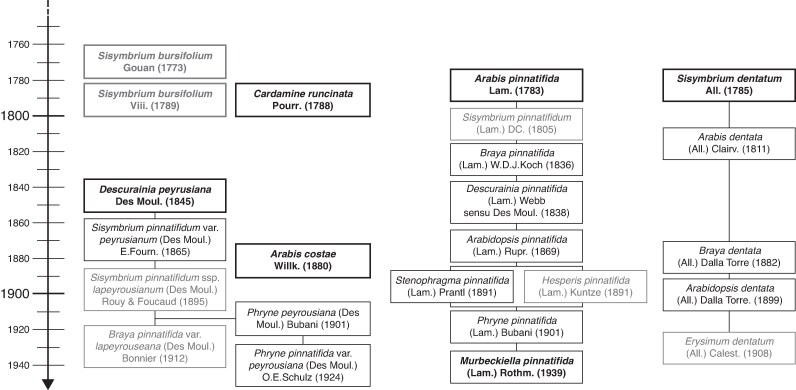
Nomenclatural history of *Murbeckiella pinnatifida*. Illegitimate names are shown in grey and heterotypic basionyms are highlighted in bold.

Despite its small size, the genus *Murbeckiella* has not been studied phylogenetically and its taxonomic history remains surprisingly chaotic. For instance, the first described species (*M. pinnatifida*) has been assigned to no fewer than 11 different genera, and up to 22 nomenclatural combinations have been proposed since its original description (*Arabis pinnatifida* Lam., 1783, p. 221; Fig. 2). The second species (*M. boryi*) was first described by Bory. The name *Cardamine heterophylla* Bory (1820, p. 6) proved illegitimate, however, and was eventually renamed by Boissier (*Cardamine boryi* Boiss., 1838, p. 9). Long considered a subspecies or variety of *M. pinnatifida*, this taxon has been assigned to no fewer than ten genera covering 24 nomenclatural combinations. Boissier (1856, p. 18) also described *Cardamine huetii* Boiss. from eastern Anatolia. A first taxonomic treatment of the whole group, for a total of four species including the earlier described *Erucastrum zanonii* Ball (1860, p. 251), was performed by Schulz (1924) who, however, used the illegitimate (superfluous) name *Phryne* Bubani (1901). Finally, Rothmaler (1939) proposed the genus *Murbeckiella* to replace *Phryne* and described a fifth species, *M. sousae* Rothm. (1939, p. 474).

Recent phylogenetic analyses of the crucifer family (Warwick *et al*., 2010; Hendriks *et al*., 2023) suggest close relationships between *Murbeckiella* and *Oreophyton* O.E.Schulz (1924). The latter genus consists of a single tetraploid species [*O. falcatum* (E.Fourn.) O.E.Schulz] that inhabits the highest peaks of East Africa (Simien and Virunga Mountains, Aberdare Range, Mount Kenya, Mount Elgon, Mount Kilimanjaro). Neither Schulz, who first described *Oreophyton*, nor Rothmaler, who proposed *Murbeckiella*, ever suggested a potential relationship between the two genera, which today make up the tribe Oreophytoneae Al-Shehbaz *et al.* (Warwick *et al*., 2010).

The currently confusing taxonomy, at either generic or tribal level, can be seen as the result of several factors, such as a potential young age, the confounding effect of polyploidy and/or important morphological plasticity, which globally impede the use of few morphological or molecular characters for species delimitation. Consequently, a comprehensive phylogenomic analysis of the recently proposed Oreophytoneae is urgently needed before undertaking any further research on this group. As a species-poor tribe, with some taxa exhibiting a small haploid genome size (1C = 0.18 pg for *M. pinnatifida* and 1C = 0.2 pg for *M. boryi*; Koch *et al*., 2012; Kiefer *et al*., 2014), the Oreophytoneae can be investigated with whole-genome sequencing (WGS) data.

The presence of both diploid and polyploid species, as well as taxa with unknown ploidy level, makes the study group suitable to test the Paralog PhyloGenomics (PPG) approach recently proposed by Walden *et al*. (2024) for targeted sequencing data. This method makes use of established pipelines for target enrichment data to first assemble all homologues of the targeted loci, detect putative polyploids and hybrids, and then reconstruct a species tree using diploids only, in which finally all homologues from polyploids and hybrids are placed using phylogenetic placement. The resulting placement patterns notably allow the distinction between different evolutionary scenarios for the emergence of polyploids: allopolyploidy, which results from hybridization between extant or extinct (ghost) lineages, and autopolyploidy, which is simply defined by ancient whole-genome duplication (WGD) within a single taxon. Importantly, the method can be applied to large datasets without prior knowledge of polyploidy and reticulate evolution of the studied taxa.

In this paper, we generated WGS data for all putative members of the Oreophytoneae, to provide the first comprehensive phylogeny of the group and to test the applicability of the PPG method. More specifically, we wanted (1) to verify the monophyly of the hitherto proposed genera *Murbeckiella* and *Oreophyton*, (2) to estimate the spatio-temporal origins of the major inferred clades, and (3) to describe several biogeographical scenarios that may explain the observed range disjunctions between the species. Finally, we propose new nomenclatural combinations to accommodate our phylogenetic results.

## MATERIALS AND METHODS

### Taxon sampling

In this study, we used 45 accessions (including 43 newly collected) encompassing all species currently assigned to the Oreophytoneae: *Murbeckiella pinnatifida* (25 accessions), *M. boryi* (7), *M. zanonii* (3), *M. omissa* (3), *M. huetii* (3), *M. sousae* (2) and *O. falcatum* (2). Overall, our sampling of the ingroup covered most of the current geographical range of the Oreophytoneae, except for the rare Moroccan populations of *M. boryi*. Whenever possible, we favoured silica-gel-dried leaf tissues from extant populations over probes from herbarium sheets (Davis *et al*., 2024); most populations of *M. pinnatifida* were indeed collected in the field during summer 2019. The following sampling permits were obtained: 001429/2019 from the Parco Nazionale Gran Paradiso, 2019-101 from the Parc national des Pyrénées, 2019-192 from the Parc national des Écrins, 2019-282 from the Parc national de la Vanoise and TREL1902817S/155 from the Ministère de la transition écologique et solidaire.

Based on previous phylogenetic studies (Warwick *et al*., 2010; Hohmann *et al*., 2015; Nikolov *et al*., 2019; Hendriks *et al*., 2023) and preliminary unpublished analyses, the following lineages–supertribes, tribes and genera have been included as outgroups: (1) lineage I, Camelinodae: Alyssopsideae (*Alyssopsis*, *Olimarabidopsis*), Arabidopsideae (*Arabidopsis*), Boechereae (*Boechera*), Camelineae (*Camelina*, *Capsella*), Cardamineae (*Cardamine*, *Nasturtium*, *Rorippa*), Descurainieae (*Descurainia*, *Tropidocarpum*), Erysimeae (*Erysimum*), Physarieae (*Physaria*), Turritideae (*Turritis*); (2) lineage II, Brassicodae: Brassiceae (*Brassica*), Thlaspideae (*Didymophysa*, *Thlaspi*); (3) lineage III, Hesperodae: Buniadeae (*Bunias*); (4) lineage IV, Arabodae: Alysseae (*Alyssum*, *Odontarrhena*), Arabideae (*Arabis*, *Dendroarabis*, *Draba*, *Pachyneurum*, *Pseudoturritis*, *Sinoarabis*, *Sciapiarabis*); (5) lineage V, Heliophilodae: Biscutelleae (*Megadenia*); and (6) basal lineage: Aethionemeae (*Aethionema*). Further information on voucher specimens can be found in Supplementary Data Tables S1 and S2.

### DNA isolation, library preparation and sequencing

In this study, we performed WGS for three mains reasons: (1) the relatively small size of the investigated genomes and the generated datasets; (2) the possibility of retrieving particular loci (e.g. plastid genome); and (3) the reusability of data after destructive sampling on herbarium specimens. DNA was extracted using a modified CTAB protocol or the NucleoSpin Plant II Mini kit for DNA (Macherey Nagel, Oensingen, Switzerland) according to the manufacturer's protocol. DNA concentration was measured with a Quantus Fluorometer (Promega, Dübendorf, Switzerland). Prior to library preparation, genomic DNA was sonicated for up to 3 min at 20 % amplitude (depending on the age of the sample) using a Q800R2 ultrasonicator (QSonica, Newtown, CT, USA). Libraries were finally prepared using the NEBNext Ultra II DNA Library Kit (New England Biolabs, Frankfurt, Germany) with AMPure XP beads (Beckman Coulter Genomics, München, Germany) according to the manufacturer's protocol but using only a third of the reaction volumes, size-selected for fragments of 550–600 bp and finally indexed. Libraries were assessed for quantity and quality using a Quantus Fluorometer (Promega, Dübendorf, Switzerland) and an Agilent 2200 Tape Station (D1000 ScreenTape, Agilent Technologies, Darmstadt, Germany) and finally sent to Novogene UK for Illumina sequencing (150 bp paired-end reads, 4 Gb data per library) on an Illumina NovaSeq 6000 sequencer (Illumina, San Diego, CA, USA). Raw Illumina sequence data are available in the European Nucleotide Archive (ENA) under accession number PRJEB85718.

### Phylogenetic analyses

We performed phylogenetic analyses on four different datasets: (1) the most complete dataset (hereafter ‘MULTI’) including multiple single-copy nuclear genes and all available accessions; (2) a diploid subset of MULTI (hereafter ‘MULTI2x’) excluding any putative polyploid accessions; (3) a dataset built upon each nuclear ribosomal sequence dataset (hereafter ‘ITS’) and (4) chloroplast DNA data (hereafter ‘PLASTID’) retrieved from the WGS shotguns.

R 4.2.2 (R Development Core Team, 2024) with the following R packages was used for phylogenetic analyses and presentation of results: ggplot2 3.5.1 (Wickham, 2016), ggpointdensity 0.1.0 (Kremer, 2019), viridis 0.6.5 (Garnier *et al*., 2024), ggpubr 0.6.0 (Kassambara, 2023), stringr 1.5.1 (Wickham, 2023), treeio 1.22.0 (Wang *et al*., 2020; Yu, 2022), ggtree 3.6.2 (Yu *et al*., 2017, 2018, Yu, 2020, 2022), BoSSA 3.7 (Lefeuvre, 2020), ggdendro 0.2.0 (de Vries and Ripley, 2024) and phytools 2.3-0 (Revell, 2024).

### Nuclear multigene tree reconstructions

To assess the evolutionary history of both diploid and polyploid lineages of Oreophytoneae, we extracted a set of single-copy loci for subsequent species tree reconstructions from all available accessions (see section Taxon sampling and Supplementary Data Table S1). Raw reads were first trimmed for quality using Trimmomatic 0.39 (Bolger *et al*., 2014) with leading and trailing minimum quality 20, sliding window quality 20 in windows of size 4, and minimum length 30. Reads from samples sequenced on multiple lanes were then merged. Target enrichment loci from the Angiosperms353 (Johnson *et al*., 2018) and Brassicaceae764 (Nikolov *et al*., 2019) bait sets, which comprise in total 1081 unique nuclear loci, were assembled using HybPiper 2.3.0 (Johnson *et al*., 2016) with bwa (Li, 2013), before retrieving assembly statistics. Based on these statistics, the *Murbeckiella sousae* sample MURBECK#024 was excluded due to low gene recovery rates (Supplementary Data Fig. S1).

Homologous gene sequences (hereafter ‘paralogues’ following terminology used by HybPiper, though these include orthologues, homeologues and paralogues) were retrieved with HybPiper. Then, allele divergence (AD) and locus heterozygosity (LH) were evaluated with HybPhaser 2.0 (Nauheimer *et al*., 2021) using minimum coverage 8, minimum allele frequency 0.1 and minimum count of alleles 3 to be regarded for assigning ambiguity. All paralogue sequences were finally aligned using MAFFT 7.520 (Katoh and Standley, 2013) with default settings followed by codon-aware realignment in MACSE 2.07 (Ranwez *et al*., 2018). TrimAl 1.2rev59 (Capella-Gutiérrez *et al*., 2009) was used to trim the alignments with default settings and maximum likelihood (ML) gene trees were reconstructed using IQ-TREE 2.2.2.4 (Minh *et al*., 2020) with 1000 ultrafast bootstrap replicates and substitution model GT + I + G. A species tree using all paralogues was reconstructed using ASTRAL-Pro 1.15.1.3 (Zhang *et al*., 2020).

The MULTI species tree consisted of 77 accessions with read mapping at 1030 genes after exclusion of 51 genes, for which >20 % of the taxa were missing (Supplementary Data Fig. S2). The Oreophytoneae encompassed a total of 44 accessions (*M. pinnatifida* [25 accessions], *M. boryi* [7], *M. zanonii* [3], *M. omissa* [3], *M. huetii* [3], *M. sousae* [1] and *Oreophyton falcatum* [2]; Supplementary Data Table S1). Based on AD and LH estimates (see Results section) and known ploidy levels from the literature (see Introduction), we finally excluded putative polyploid taxa to retain 58 diploid accessions. Gene trees for the diploid dataset were reconstructed with RAxML 8.2.12 (Stamatakis, 2014) using 1000 rapid bootstrap inferences followed by a thorough ML search with substitution model GTRCAT, and then combined in the MULTI2x species tree, including 39 samples of Oreophytoneae (*M. pinnatifida* [25 accessions], *M. boryi* [7], *M. zanonii* [3], *M. omissa* [3] and *M. sousae* [1]; Supplementary Data Table S1).

Phylogenetic placement of homologues from putative polyploid samples into the MULTI2x species tree was conducted following Walden *et al*. (2024). All homologues were placed into their respective gene trees (rooted using *Aethionema saxatile* as outgroup) using the evolutionary placement algorithm implemented in RAxML with substitution model GTRCAT. Genes with >10 % of diploid samples missing and genes with >10 % of diploid samples having multiple homologues, such as those retained from the At-α WGD shared by all Brassicaceae, were excluded from further assessment. For the remaining 826 genes, we analysed the likelihood–weight ratios (lwrs) for the placement of each homologue into the branches (edges) of gene trees and the corresponding edges in the MULTI2x species tree. For each sample, we calculated cumulative lwr across all homologues and genes for each edge and normalized by the sum of lwr, thereby accounting for missing data. Finally, hierarchical clustering on the resulting placement patterns was conducted, similar placement patterns indicating a likely shared evolutionary history of the respective samples.

### ITS tree reconstruction

To assess the phylogenetic position of low-quality accessions that were excluded in the MULTI/MULTI2x datasets and to take advantage of the Phylogenetics Tool in BrassiBase (Koch *et al*., 2012; Kiefer *et al*., 2014), we reconstructed the nuclear ribosomal cistrons, comprising the internal transcribed spacers and the 5.8S gene (hereafter ‘ITS sequences’). Illumina reads mapping against selected ITS sequences of lineage I, Camelinodae (*Arabidopsis-*Arabidopsideae, *Capsella-*Camelineae, *Cardamine*-Cardamineae) and lineage IV, Arabodeae (*Arabis-*Arabideae) were filtered using BBMap 35.45 (Bushnell, 2014) and reference-guided assembled into 95 % consensus sequences using Geneious R9.1.8 (https://www.geneious.com) with default settings. These ITS sequences were then aligned with MAFFT 7.520 (Katoh and Standley, 2013) using default settings and the alignment was trimmed using trimAl in strict mode (Capella-Gutiérrez *et al*., 2009). An ML tree was finally reconstructed with RAxML 8.2.12 (Stamatakis, 2014) using 1000 rapid bootstrap inferences followed by a thorough ML search with substitution model GTRCAT. The complete ITS dataset finally consisted of a total of 70 accessions, including 45 Oreophytoneae (*Murbeckiella pinnatifida* [24 accessions], *M. boryi* [7], *M. zanonii* [3], *M. omissa* [3], *M. huetii* [4], *M. sousae* [2] and *Oreophyton falcatum* [2] and 25 outgroups; Supplementary Data Table S1). The phylogram was rooted with Aethionemeae (*Aethionema*) using FigTree 1.4.4 (http://tree.bio.ed.ac.uk/software/figtree/).

### Plastid tree reconstruction

To uncover putative cytonuclear incongruence and to take advantage of the large plastid-based dataset available for dating Brassicaceae lineages, 43 Oreophytoneae chloroplast genomes (*M. pinnatifida* [24 accessions], *M. boryi* [7], *M. zanonii* [3], *M. omissa* [3], *M. huetii* [3], *M. sousae* [2] and *O. falcatum* [1]; Supplementary Data Table S1) were assembled from the newly generated raw sequence data using NovoPlasty 4.2.1 (Dierckxsens *et al*., 2016). For divergence time estimation (see next paragraph), we added 21 fully assembled plastomes from across the major Brassicaceae lineages as outgroups for a final PLASTID dataset of 64 accessions (see Taxon sampling and Supplementary Data Table S1). The coding sequences of 78 protein-coding genes and four ribosomal RNA genes were then selected for phylogenetic reconstruction. Trans-spliced protein-coding gene *rps*12 was split into its three parts for easier alignment; thus, the total number of alignments was 84. Gene sequences from newly assembled *Murbeckiella* and *Oreophyton* plastids and those from outgroups were obtained by aligning the respective sequence from *Arabidopsis thaliana* (NCBI accession AP000423) to the other sequences using MAFFT 7.520 (Katoh and Standley, 2013) with parameters --6merpair --adjustdirection --keeplength --addfragments, which aligns a reference sequence to any number of other sequences and retains only the aligned parts. All alignments were then manually checked (see Supplementary Data File S1 for complete alignment). Gene sequences were concatenated using IQ-TREE 2.2.2.4 (Minh *et al*., 2020), then partitioning scheme and substitution models were determined. An ML tree with ultrafast bootstrapping was constructed using resampling for partitions and sites within partitions (option --sampling GENESITE).

### Divergence time estimations

Divergence time estimation was performed on the PLASTID dataset in Beast 2.7.7 (Bouckaert *et al*., 2019). The nine partitions were implemented with their best substitution model (Supplementary Data File S1) with independent clock models and linked trees for all partitions. We used a lognormal relaxed clock (Drummond *et al*., 2006; Douglas *et al*., 2021) and tree model birth-death (Gernhard, 2008). Two secondary calibration points were extracted from the literature (Walden *et al*., 2020) by fitting a lognormal distribution on the respective node ages of a Brassicaceae plastid divergence time estimation using R packages ape 5.8 (Paradis and Schliep, 2018), phytools 2.1-1 (Revell, 2024), scales 1.3.0 (Wickham *et al*., 2023) and fitdistrplus 1.1-11 (Delignette-Muller and Dutang, 2015). The calibration points were set on the root by constraining the stem age of core Brassicaceae (all but *Aethionema saxatile*), i.e. the split between *Aethionema* and the rest of the family, using a lognormal distribution with meanlog 3.39442165 and sdlog 0.09822607, and the crown age of the same group was set to meanlog 3.22063376 and sdlog 0.08884421. Four independent runs of chain length 10^7^ were run, logging every 50 000 generations. The first 20 % of each run were discarded as burn-in, and the remaining trees were combined using logcombiner and annotated using treeannotator to obtain a final time-calibrated plastid tree.

## RESULTS

### Sequencing data and ploidy level estimations

We recovered high numbers of genes from the Angiosperms353 and Brassicaceae764 target enrichment datasets using WGS data despite a lower number of reads mapped to the target loci. Generally, around 300 000 mapped reads were sufficient in our dataset to cover at least 900 genes over at least 50 % of the target length. The samples with the lowest gene recovery rates were generally sequenced from herbarium material (Supplementary Data Fig. S1).

We used AD and LH as a proxy for polyploid and/or hybrid detection by setting the minimum LH to 90 and AD to 2 following the thresholds set by Walden *et al*. (2024) for detecting polyploids and hybrids in tribe Arabideae. Accordingly, we estimated a diploid origin for *M. zanonii* and *M. sousae*. The levels of AD and LH detected in these two species indeed fell within the range of values found in the diploids *M. pinnatifida* and *M. boryi* (AD_max_ = 1 %, LH_max_ = 75 %; Supplementary Data Fig. S3). By contrast, all but one sample of polyploid *M. huetii* and *O. falcatum* displayed higher values of AD and LH. The unexpectedly low estimations (AD ∼ 1 %, LH ∼ 75 %) detected in one sample of *M. huetii* (MURBECK#045) may be explained by insufficient coverage, with only 168 216 mapped reads (Supplementary Data Fig. S1). Finally, the levels of AD and LH calculated for diploid *M. omissa* fell between these two extremes (1 % > AD > 2 %, 75 % > LH > 90 %). However, as *M. omissa* still fell below the threshold for polyploids and the species had been reported to be diploid, we did not exclude the samples from the MULTI2x dataset.

Similar to gene recovery rate, the number of paralogues detected and assembled by HybPiper was low for samples with few mapped reads, though also here as few as 680 000 mapped reads were sufficient to detect paralogues in >100 loci (Supplementary Data Fig. S4). HybPiper detected between 0 and 567 paralogues among the samples (*M. huetii* MURBECK#45 and *Descurainia sophia* S1307, respectively). Generally, diploid samples had fewer paralogues (2–113, median 24, mean 22.95) than polyploids (0–567, median 110, mean 149.2).

### Phylogenetic relationships

**Fig. 3.**
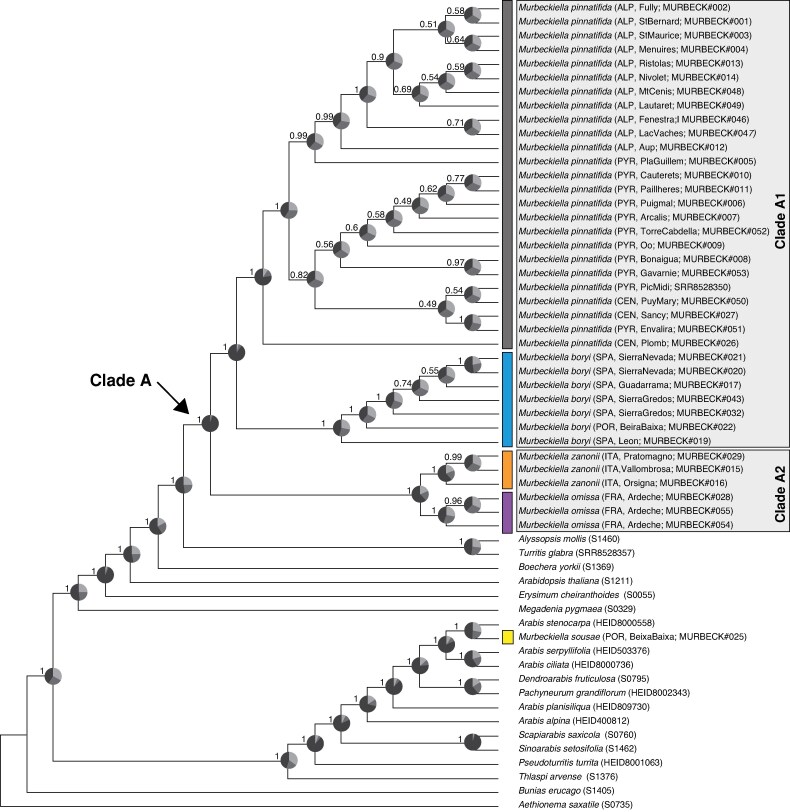
‘Diploid’ tree: ASTRAL-Pro species tree based on 1030 gene trees reconstructed from paralogues of ‘diploid’ samples only. Pie charts illustrate quartet support for the major, first alternative and second alternative topology at each branch (dark grey, medium grey and light grey, respectively); posterior probabilities are given above the branches. The taxa are coloured as in Fig. 1.

The MULTI2x species tree revealed the polyphyly of the currently circumscribed Oreophytoneae: (1) *M. pinnatifida*, *M. boryi*, *M. zanonii* and *M. omissa* forming a highly supported monophyletic diploid clade A (posterior probability for the main topology p1 = 1, quartet support for the main topology q1 = 0.97) sister to a clade assigned to Turritideae; (2) *M. sousae* being phylogenetically distant from clade A and sister to *Arabis stenocarpa* (Arabideae; Fig. 3). Within clade A, subclade A1 (*M. pinnatifida*, *M. boryi*) was sister to subclade A2 (*M. zanonii, M. omissa*). Importantly, while quartet support was low within the species, the respective branches separating the subclades and species had high support (Fig. 3).

**Fig. 4.**
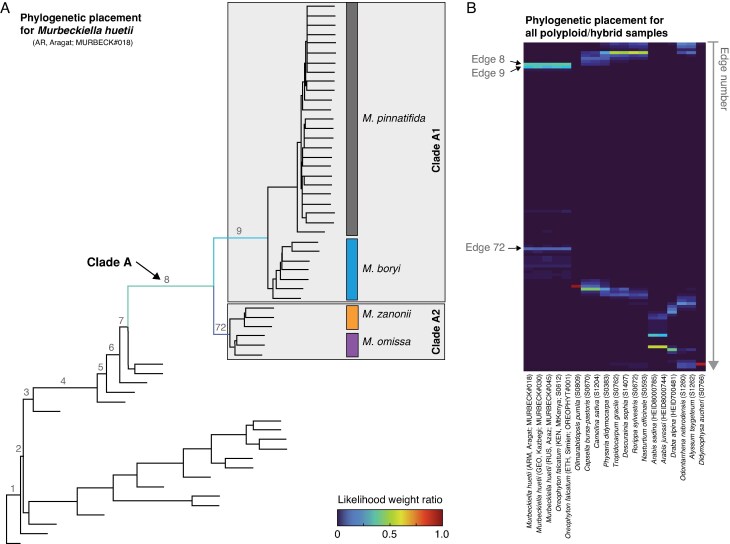
Phylogenetic placement of ‘polyploids’. (A) Diploid tree with branches coloured according to placement of paralogues from a *Murbeckiella huetii* sample and (B) cumulative likelihood-weight ratios over all gene copies from putative ‘polyploid/hybrid’ samples.

The phylogenetic placement of the respective paralogues of tetraploid *O. falcatum* and octoploid *M. huetii* into the MULTI2x topology produced identical results (Fig. 4A). In each case, the branches with the highest cumulative lwr were the branch of clade A and the branch of subclade A1 (*M. pinnatifida*, *M. boryi*). The analysis of the normalized branch-specific lwr further showed almost equal proportions of paralogues mapping to the two putative ancestral edges for all five samples of this group (Fig. 4B). In the MULTI species tree, these two polyploids formed a moderately supported clade B (p1 = 1, q1 = 0.52) nested between subclades A1 and A2, though this position was not highly supported (p1 = 1, q1 = 0.45; Supplementary Data Fig. S5).

The ITS topology showed some incongruence to the one inferred from the MULTI dataset, with clade B (*O. falcatum*, *M. huetii*) sister to clade A2 (*M. zanonii*, *M. omissa*), though this grouping only had a low bootstrap support (BS) of 37. Within tribe Arabideae, *M. sousae* was sister to *Dendroarabis fruticulosa* (Supplementary Data Fig. S6).

Finally, polyploid clade B (*O. falcatum*, *M. huetii*) was sister to clade A in the PLASTID tree, in which subclade A2 (*M. zanonii*, *M. omissa*) was nested within subclade A1 (*M. pinnatifida*, *M. boryi*), with high BS of 94–100 for the relevant branches (Supplementary Data Fig. S7). As opposed to the MULTI species tree, clade AB (Oreophytoneae excluding *M. sousae*) was sister to a clade assigned to Arabidopsideae and Camelineae (instead of Turritideae).

Together, our findings for the polyploid *M. huetii* and *O. falcatum* indicate an allopolyploidization event shared by the two species for several reasons: (1) cytonuclear discordance is shown by conflicting topologies between plastid and nuclear trees; (2) support for the position of this clade was low in the MULTI tree, indicating conflicting signals in the underlying gene trees; and (3) the placement pattern from phylogenetic placement indicates two edges as the potential ancestors for the samples with almost even lwr proportions, in line with a hybrid origin of the clade.

### Divergence time estimations

**Fig. 5.**
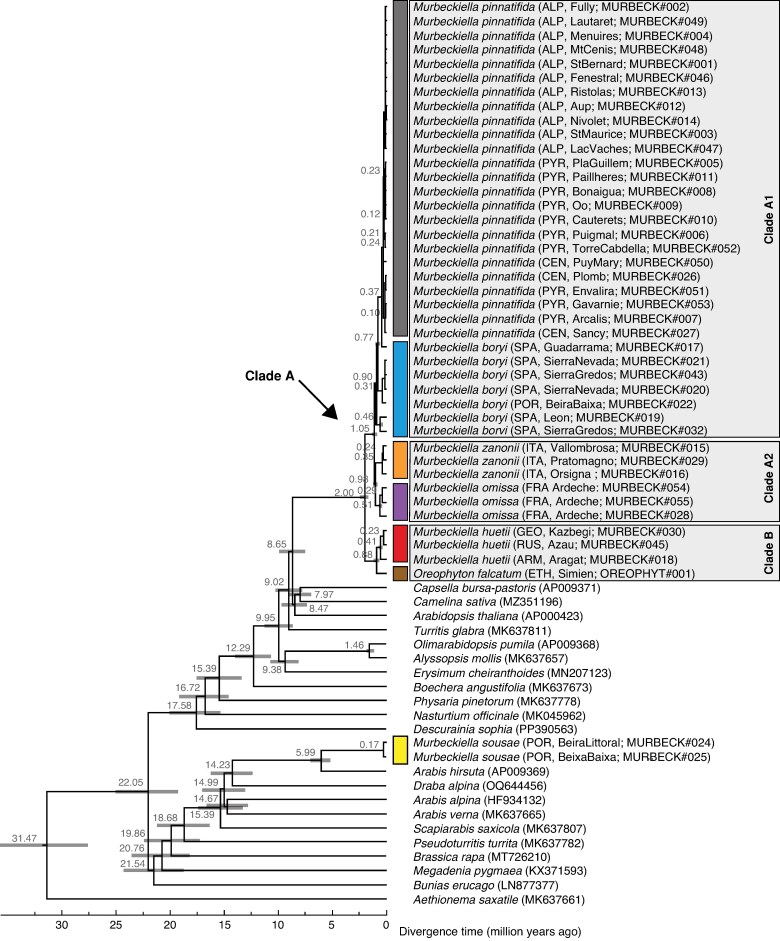
Divergence time estimation with BEAST based on 82 plastid genes. Age estimates are given above the nodes; 95 % highest posterior density intervals are shown as node bars. The taxa are coloured as in Fig. 1.

In the time-calibrated PLASTID tree, the polyploid clade B diverged from the sister diploid clade A ∼2 million years ago (mya; 95 % highest posterior density [HPD] 1.63–2.40; Fig. 5). The subsequent divergence between subclades A1 and A2 was dated at ∼1 mya (95 % HPD 0.86–1.26), an age that roughly coincided with the diversification of the polyploid clade B. Within Arabideae, the split between *M. sousae* and the closest relative included in the analyses (*Arabis hirsuta*) was estimated at ∼6 mya (95 % HPD 5.10–6.96). Divergence times of the Brassicaceae backbone and the tribes included in the PLASTID tree were generally in line with those estimated in previous studies (Hohmann *et al*., 2015; Walden *et al*., 2020; Hendriks *et al*., 2023).

## DISCUSSION

### Applicability of the PPG approach to WGS

The availability of constantly increasing high-quality next-generation sequence data provides the opportunity to combine existing with newly generated phylogenomics datasets, to unravel the evolutionary history of new lineages. For the Brassicaceae family, large target enrichment datasets are meanwhile available for hundreds of taxa (Nikolov *et al*., 2019; Hendriks *et al*., 2023) and can be used as outgroups for detailed studies in specific tribes or genera. WGS data have been integrated into target enrichment analyses previously (Balant *et al*., 2025), and it has been shown that successful recovery of targeted loci mostly depends on sequencing depth. We observed the same in our Oreophytoneae dataset and excluded the sample with fewest mapped reads and lowest gene recovery rate.

Despite lower coverage in WGS samples, ploidy detection using heterozygosity and sequence divergence worked well for most samples with known ploidy. However, we found that the number of mapped reads required for successful ploidy detection was much higher (more than doubled) than for gene recovery. Interestingly, while the number of genes with paralogues detected was low in WGS samples, phylogenetic placement of polyploid Oreophytoneae showed a nearly even distribution of homologues into two ancestral branches, indicating that high numbers of paralogues are not necessary to detect hybrid ancestry using PPG. This is most relevant for established polyploids undergoing the process of genome downsizing termed diploidization, in particular when using target enrichment loci, which generally contain conserved single-copy genes (De Smet *et al*., 2013; Li *et al*., 2016; Walden and Schranz, 2023). Finally, our results of phylogenetic placement for polyploid clade B is fully congruent with both the basal position of the clade supported by the maternally inherited plastid and the position nested between diploid subclades A1 and A2 supported by nuclear phylogenies.

### Natural history of Oreophytoneae

Unless stated otherwise, the discussion relies on the robust and fully resolved species trees inferred from the MULTI/MULTI2x datasets. Our phylogenetic analyses support for the first time the polyphyly of *Murbeckiella* in its current taxonomic circumscription, and its paraphyly with respect to *Oreophyton* (Fig. 3, Supplementary Data Figs S5–S7). Indeed, in the respective MULTI/MULTI2x species trees, *M. sousae* is sister to the Iberian endemic *Arabis stenocarpa* (Arabideae, sect. *Turritella*; Fig. 3, Supplementary Data Figure S5), whereas the ITS topology supports a sister position of *M. sousae* with *D. fruticulosa* (tribe Arabideae). While the limited sampling of the PLASTID tree does not provide a close relative, it also places the species in the *A. hirsuta* clade (for this clade refer to Karl and Koch, 2013, 2014) within Arabideae. The systematic exclusion of *M. sousae* from the tribe Oreophytoneae and its inclusion in the Arabideae has never been proposed so far.

*Murbeckiella sousae* differs from the remaining species currently ascribed to the genus by a set of morphological characters: the cauline leaves are subentire to dentate but barely auriculate (versus prominently auriculate and always pinnatifid in other species of *Murbeckiella*), while the basal ones are always lyrate-pinnatifid (versus subentire to pinnatifid); the flowers and the siliques are larger in size; the seeds are circumferentially winged; and the indumentum, consisting of both simple and branched trichomes, is very sparse (Rothmaler, 1939; A. Guggisberg, pers. obs.). In line with our findings, these phenotypic differences led Rothmaler (1939) to express some doubts before including his new species (*M. sousae*) in *Murbeckiella*. Our phylogenetic analyses confirm Rothmaler's hesitations and the affiliation of *M. sousae* to Arabideae. However, a precise taxonomic status of the presumably diploid *M. sousae* cannot be stated with confidence before a complete taxonomic revision of the highly polyphyletic genus *Arabis* is completed (Karl and Koch, 2013; Hendriks *et al*., 2023; Walden *et al*., 2024).

The phylogenetic exclusion of *M. sousae* does not make *Murbeckiella* monophyletic. Indeed, the highly supported and well-embedded polyploid clade B overall makes *Murbeckiella* paraphyletic with respect to *Oreophyton* (Supplementary Data Fig. S5). This unexpected result entails new taxonomic circumscriptions, both at the generic and tribal level, and has important nomenclatural consequences that are appended to this Discussion. By lumping two genera into one, we must keep the name of the first described and validly published taxonomic entity. As *Oreophyton* (Schulz, 1924) predates *Murbeckiella* (Rothmaler, 1939), it has priority and our newly defined genus should bear this name. By the same token, *Oreophyton* loses its status of endemic afroalpine monotypic genus (cited in Brochmann *et al*., 2022).

Before the description of tribe Oreophytoneae based on molecular grounds (Warwick *et al*., 2010), the affinity between *Oreophyton* and *Murbeckiella* has never been proposed. In his impressive taxonomic treatment of Sisymbrieae, Schulz (1924) assigned *Oreophyton* and *Murbeckiella* (then under ‘*Phryne*’) to different subtribes – Pachycladineae (p. 181) and Sisymbriineae (p. 45), respectively – on the basis of fruit size (p. 19). These preliminary, highly artificial circumscriptions had undesirable consequences, for it prevented Schulz from directly comparing *Phryne* with his new genus *Oreophyton*. Overall, species of *Phryne* (= *Murbeckiella*) show rather long (2–8 cm) siliques on proportionally short pedicels (ratio fruit/pedicels ∼2–10) with uniseriate seeds, while the monospecific *Oreophyton* exhibits short (1–2 cm) siliques on proportionally long pedicels (ratio fruit/pedicels 0.3–2) with biseriate seeds (H. Schnorf, 2021, unpubl. res., Institute of Integrative Biology, ETH Zurich, Switzerland). Since convergence (homoplasy) is pervasive in Brassicaceae (Al-Shehbaz, 2012), taxon delimitation should always integrate multiple lines of evidence in this plant family.

Our phylogenetic analyses support a new taxonomic definition of the genus *Oreophyton* (six species, excluding *M. sousae*) that makes Oreophytoneae monogeneric. Depending on the dataset used, the strongly supported clade AB (newly defined Oreophytoneae*-Oreophyton*) is either sister to clades assigned to Turritideae and Alyssopsideae (p1 = 1, q > 0.97, MULTI/MULTI2x species trees; Fig. 3, Supplementary Data Fig. S5; BS < 50, ITS tree, Supplementary Data Fig. S6), or Arabidopsideae and Camelineae (BS = 99, PLASTID tree, Supplementary Data Fig. S7). Such cytonuclear discordance between multi- and single-locus phylogenies corroborates earlier work by Hendriks *et al*. (2023) and highlights complex evolutionary pathways involving extensive hybridization and WGD between divergent lineages such as supertribes Camelinodae and Brassicodae (Guo *et al*., 2020). To overcome misleading interpretations due to reticulate evolution, we therefore privilege the MULTI/MULTI2x topologies (Fig. 3, Supplementary Data Fig. S5) when formulating preliminary biogeographical hypotheses.

The ancestor of Oreophytoneae most likely originated in Anatolia or adjacent Levant. Indeed, all sister lineages of clade AB – the newly defined Oreophytoneae*-Oreophyton* – in the respective species trees predominantly entail Irano-Turanian elements (Fig. 3, Supplementary Data Fig. S5): (1) apart from the cosmopolitan *Turritis glabra*, tribe Turritidae contains only one species, *T. laxa*, which occurs in the Middle East and in the Balkans; (2) taxa of tribe Alyssopsideae are concentrated in Iran and neighbouring countries of Central Asia with few range extensions to Transcaucasus (*Alyssopsis*) and China (Olimarabidopsis; Koch *et al*., 2012; Kiefer *et al*., 2014). In line with our hypothesis, the Irano-Turanian region was repeatedly proposed as an important centre of Brassicaceae diversification (Koch and Kiefer, 2006; Franzke *et al*., 2009; Ansell *et al*., 2011; Karl and Koch, 2013; Mohammadin *et al*., 2017; Ali *et al*., 2019).

Based on the observation of the current disjunct geographical range of *Murbeckiella* in Eurasia, Rothmaler (1939) first proposed the genus to be a Tertiary relict of a previously widespread ancestor. This hypothesis is contradicted by our molecular dating analyses, which suggest instead a much more recent diversification of the group during the Pleistocene (∼2 mya). At that time, repeated cycles of glaciations and deglaciations (Dynesius and Jansson, 2000) caused recurrent shifts in alpine vegetation across the globe, with range expansions during cold and dry glacial periods, followed by contractions during warm and mostly humid interglacial phases (Flantua and Hooghiemstra, 2018). The rhythmic fragmentations of formerly continuous distributions (also known as vicariance) coupled with instances of long-distance dispersal across the topographical barriers finally shaped contemporary mountain diversity (Hewitt, 2000, 2004; Schmitt, 2007, 2009; Varga and Schmitt, 2008). Intercontinental exchanges between Europe and Asia were possible during the cold, glacial periods, when land bridges formed across the Sea of Marmara and the Bosporus (Ansell *et al*., 2011 and references therein).

Our phylogenetic analyses support a common origin for the morphologically close species *M. pinnatifida* and *M. boryi* (subclade A1, p1 = 1, q1 > 0.9; Fig. 3, Supplementary Data Fig. S5). In previous taxonomic treatments, *M. boryi* has been repeatedly treated as a subspecies or variety of *M. pinnatifida*, because only minor morphological differences could help discriminate these taxa. *Murbeckiella boryi* usually exhibits fewer (two to five) cauline leaves with fewer (one to three) but broader (up to 3.5 mm wide) pairs of leaf lobes than *M. pinnatifida* (six to ten leaves with four to six pairs of lobes that are up to 1.5 mm wide; Schulz, 1924; Rothmaler, 1939; Luceño, 1997), but overall the taxonomic distinction between the two species remains elusive in the absence of geographical information. In line with our molecular dating, the strong phenotypic similarity between *M. pinnatifida* and *M. boryi* is likely the result of their very recent divergence.

The inclusion of multiple accessions of *M. pinnatifida* and *M. boryi*, covering most of their current distributional range, suggests some preliminary (phylo)geographical patterns. In the MULTI2x species tree (Fig. 3), *M. pinnatifida* forms two well-supported subclades, one encompassing all Alpine accessions, the other most of the accessions from the Massif Central and the Pyrenees. Since the Alpine clade of *M. pinnatifida* always showed a derived phylogenetic position in our analyses, we can hypothesize a recent colonization of the western Alps from a more southern centre of diversification in the Pyrenees or Massif Central (Fig. 1). Similarly, accessions of *M. boryi* from the Sierra Nevada occupy a derived position with respect to accessions from the rest of Iberia in the MULTI2x species tree (Fig. 3), suggesting a stepwise diversification from northwestern Iberia over the Central System to the Baetic System. More extensive analyses, including accessions from the Atlas Mountains, would help to resolve the phylogeographical scenario for the recently diverged subclade A1.

Our study strongly supports the specific status of *M. omissa* proposed by Chéron (2020) to accommodate the taxonomic differentiation of *Murbeckiella* from the Cévennes. In all our phylogenies, accessions of *M. omissa* indeed form a monophyletic group that is sister to the *M. zanonii* clade (subclade A2, p1 = 1, q1 = 0.62/0.66 in MULTI2x/MUTLI; Fig. 3, Supplementary Data Fig. S5). Populations from the eastern Massif Central have long been confused with *M. zanonii*, which was described from the Apennines (summarized in Chéron, 2020). However, individuals from the former (*M. omissa*) exhibit a lower number of lobes on the cauline leaves (8–12 versus 11–15), shorter pedicels (in flower: 4–6 versus 6–15 mm; in fruit: 4.5–12 versus 8–22 mm) and shorter siliques (10–20 versus 27–41 mm) compared with *M. zanonii* (Chéron, 2020). This taxonomic confusion had led different authors to treat the Cévennes populations as either ancient, indigenous relicts (Tison and de Foucault, 2014), or recent, non-native introductions (Thévenot *et al*., 2022) of *M. zanonii*. Our phylogenetic results reject both hypotheses and call for the recognition of a geographically highly restricted endemic taxon. Presently, *M. omissa* is listed as Vulnerable (VU) in the IUCN Red List of Threatened Species of France under the erroneous mention of *M. zanonii* (UICN France *et al*., 2018). Given its limited geographical range (76 km^2^), the small size (1–15 m^2^) of its populations and the high risk of fire in its natural habitat, a reassessment as an Endangered (EN) or Critically Endangered (CR) species should be urgently reconsidered (Chéron, 2020).

Despite its restricted range, *M. omissa* exhibits a high level of heterozygosity (Supplementary Data Fig. S3), in line with the obligate outcrossing breeding system recorded for this taxon (Chéron, 2020). Two additional, non-mutually exclusive hypotheses resulting from our analyses may be put forward to explain this pattern: (1) *M. omissa* represents a relict species that did not undergo any major range contractions during warm, interglacial phases and therefore retained most of its original genetic diversity; (2) *M. omissa* is the result of some homoploid hybridization or ancient introgression events, which caused a significant increase in allelic richness, but simultaneously affected its fitness. The fact that the few seeds being produced usually abort and the species mainly propagates clonally (Chéron, 2020) supports a hybrid origin for *M. omissa*. More detailed genetic investigations are needed to decide between these different assumptions.

Our analyses always support a common origin for the respective polyploid taxa, *O. falcatum* and *M. huetii* (clade B, Fig. 4, Supplementary Data Figs S5–S7). However, the phylogenetic position of the inferred clade B greatly varies depending on the dataset used, being embedded between subclades A1 and A2 in the MULTI species tree (Supplementary Data Fig. S5), or sister to clade A in the PLASTID phylogram (Supplementary Data Fig. S7). This topological incongruence suggests a hybrid origin for these two taxa. When analysing the placement of these polyploids in the diploid tree, paralogues of *O. falcatum* and *M. huetii* indeed either fall with subclade A1 (*M. pinnatifida*/*M. boryi*) or at the base of clade AB (newly defined Oreophytoneae*-Oreophyton*; Fig. 4). This pattern is best explained by allopolyploidization between the most recent ancestor of subclade A1 and an (extinct) sister lineage of clade AB.

Because the Irano-Turanian region is hypothesized to constitute the cradle of Oreophytoneae, the ancestor of *O. falcatum* most likely originated in the Middle East before recently dispersing to Africa ∼1 mya (95 % HPD 0.64–1.13; Figs 1 and 5). This result is in line with a recent review by Brochmann *et al*. (2022), which suggests that most Afroalpine lineages actually colonized eastern Africa via long-distance dispersal from remote temperate regions during the last ten million years. *Arabis alpina*, for instance, colonized eastern Africa multiple times from Anatolia during the early Middle Pleistocene (2.7 mya; Koch *et al*., 2006; Assefa *et al*., 2007; Ansell *et al*., 2011).

Similarly, the ancestor of *M. huetii* is likely to have originated in the Middle East before reaching the Caucasus very recently, ∼1 mya (95 % HPD 0.64–1.13; Figs 1 and 5). During the cold, glacial periods of the Pleistocene, cold-adapted plants could indeed expand their range from the Middle East to the Caucasus and Near East mountain systems range via the highest peaks of Anatolia (the so-called ‘Anatolian diagonal’ above 2000 m a.s.l.; Ansell *et al*., 2011 and references therein). A further spread into Asia was, however, likely hampered by the rise of the Caspian Sea and the steady aridification of Central Asia during the warm, interglacial phases (Manafzadeh *et al*., 2017). Overall, a global biogeographical analysis of the newly defined Oreophytoneae, including a more comprehensive geographical sampling of the group, is highly needed before any further conclusions.

### Taxonomic conclusions and nomenclatural changes

Our WGS-based phylogenetic analyses evidenced for the first time the polyphyly of *Murbeckiella*, with *M. sousae* placed in a clade of distinct tribe Arabideae, and its further paraphyly with respect to *O. falcatum*. If the taxonomic status of *M. sousae* is awaiting future taxonomic revision of the polyphyletic genus *Arabis* (Walden *et al*., 2024), all other species hitherto placed into *Murbeckiella* should be integrated in a newly defined genus, *Oreophyton*. Indeed, in our MULTI analyses all accessions of *Oreophyton* are embedded in a well-supported clade AB (Supplementary Data Fig. S6). From a nomenclatural viewpoint, the new genus corresponding to clade AB, and resulting from the lumping of two genera into one, should bear the name of the first-described and validly published taxonomic entity, here *Oreophyton* (Schulz, 1924). *Murbeckiella* was indeed published later (Rothmaler, 1939) to provide a legitimate generic name for the species assigned by Schulz to the illegitimate *Phryne* Bubani (Schulz, 1924).

### *Oreophyton* O.E.Schulz (emendatio)

Type species: *Oreophyton falcatum* (E.Fourn.) O.E.Schulz

Perennial herbs, usually with simple and multifurcate hairs (branched trichomes); basal leaves (sub)entire or pinnatifid; cauline leaves lacking (*O. falcatum*) or sessile, pinnatifid and auriculate; flowers forming an ebracteate raceme; sepals ascending; petals white or reddish; stigma entire; ovules 20–60; fruit a terete (round in cross-section) or latiseptate (compressed parallel to the septum) silique; seeds uniseriate or biseriate (*O. falcatum*), usually not mucilaginous; cotyledons with radicle along the edge, or resting on one side (incumbent cotyledons).

**Distribution.** N and E Africa, Caucasus, Europe.

**Remark.** The newly circumscribed genus *Oreophyton* corresponds to the description of the Tribe Oreophytoneae Al-Shehbaz *et al.*, excluding *Murbeckiella sousae* Rothm., which exhibits lyrate-pinnatifid basal leaves and subentire-to-dentate cauline leaves and should be newly assigned to tribe Arabideae.

Key to the species of *Oreophyton* O.E.Schulz

1. Silique with biseriate seeds; cauline leaves absent ***O. falcatum***

1. Silique with uniseriate seeds; cauline leaves present 2

2. Cauline leaves with 1–3 pairs of lobes 3

2. Cauline leaves with 4–7 pairs of lobes 4

3. Fruit pedicels erecto-patent; plant from Iberian and Atlas Mtns. ***O. boryi***

3. Fruit pedicels spreading or reflexed; plant from Caucasus ***O. huetii***

4. Flower pedicels 1.5–3 mm; fruit pedicels 3–5 mm, erecto-patent ***O. pinnatifidum***

4. Flower pedicels 4–15 mm; fruit pedicels 4.5–22 mm, spreading or reflexed 5

5. Plant (3−)7–20(−23) cm tall; basal leaves (sub)entire; siliques (0.7−)1–2(−2.5) cm long; plant from Massif Central ***O. omissum***

5. Plant (12−)25–45(−50) cm tall; basal leaves pinnatifid; siliques (2.0−)2.7–4.1(−5.0) cm long; plant from the Apennines ***O. zanonii***

Taxonomic combinations in *Oreophyton* O.E.Schulz

**1. *Oreophyton boryi*** (Boiss.) Guggisberg & Mansion, **comb. nov.** ≡ *Cardamine heterophylla* Bory, Ann. Gén. Sci. Phys. 3: 6. 1820, *nom. illeg.* ≡ *Cardamine boryi* Boiss., Elench. Pl. Nov.: 9. 1838. ≡ *Arabis boryi* (Boiss.) Boiss., Voy. Bot. Espagne, 2: 26. 1839. ≡ *Descurainia boryi* (Boiss.) Des Moul., Actes Acad. Roy. Sci. Bordeaux 7(3): 357. 1845. ≡ *Sisymbrium pinnatifidum* var. *boryanum* E.Fourn. Recherches Anat. Taxon. Fam. Crucifer.: 99. 1865. ≡ *Sisymbrium boryi* (Boiss.) Boiss., Fl. Orient. [Boissier] 1: 958. 1867, *in not.* ≡ *Sisymbrium pinnatifidum* var. *heterophyllum* Pau, Bol. Soc. Aragonesa Ci. Nat. 8: 111. 1909, *nom. illeg*. ≡ *Braya pinnatifida* var*. boryi* (Boiss.) Bonnier, Fl. Compl. France, Suisse & Belg. 1: 70. 1912, *nom. illeg*. ≡ *Stenophragma pinnatifidum* var. *boryi* (Boiss.) Cout., Fl. Portugal: 254. 1913, *nom. illeg*. ≡ *Phryne boryi* (Boiss.) O.E.Schulz, Pflanzenr. (Engler) 4, 105(86): 172. 1924. ≡ *Sisymbrium pinnatifidum* subsp. *boryi* (Boiss.) Font Quer, Cavanillesia 3: 58. 1930. ≡ *Phryne pinnatifida* subsp. *boryi* (Boiss.) Maire, Cat. Pl. Maroc 2: 275. 1932. ≡ *Murbeckiella boryi* (Boiss.) Rothm., Bot. Not. 1939: 469. 1939. ≡ *Murbeckiella pinnatifida* subsp. *boryi* (Boiss.) Maire, Cat. Pl. Maroc 4: 1003. 1941.

= *Arabis carpetana* Willk., Prodr. Fl. Hispan. 3(4): 822. 1880.

= *Cardamine resedifolia* var. *longisiliqua* Font Quer, Cavanillesia 1: 71. 1928. ≡ *Sisymbrium pinnatifidum* var*. longisiliqua* (Font Quer) Font Quer, Cavanillesia 3: 58. 1930. ≡ *Phryne pinnatifida* var. *longisiliqua* (Font Quer) Maire, Cat. Pl. Maroc 2: 275. 1932. ≡ *Murbeckiella pinnatifida* var. *longisiliqua* (Font Quer) Maire, Cat. Pl. Maroc 4: 1003. 1941.

= *Phryne glaberrima* Rothm., Bol. Real Soc. Esp. Hist. Nat. 34: 148. 1934. ≡ ‘*Braya glaberrima*’ Rothm., Bol. Real Soc. Esp. Hist. Nat. 34: 148. 1934, *nom. inval.* ≡ *Murbeckiella glaberrima* (Rothm.) Rothm., Bot. Not. 1939: 472. 1939.

 = *Murbeckiella boryi* subsp*. herminii* Rivas Mart., Anales Real Acad. Farm. 47: 475. 1981. ≡ *Murbeckiella pinnatifida* subsp*. herminii* (Rivas Mart.) Greuter & Burdet, Willdenowia 15: 68. 1985.

  **Distribution.** Iberian Peninsula (notably Cantabrian Mountains, Iberian and Central System, Sierra Nevada) and Moroccan Rif.

  **Remark.** Despite the fact that Fournier's combination (*Sisymbrium pinnatifidum* var. *boryanum*, 1865) contradicts the Recommendation 24B.2 of the International Code of Nomenclature, his variety *boryanum* remains legitimate (a new name at a new rank, using *Cardamine boryi* Boiss. as a replaced synonym). Consequently, the later combinations at varietal rank (i.e. var. *boryi* and var. *heterophyllum*) become illegitimate.

**2. *Oreophyton falcatum*** (E.Fourn.) O.E.Schulz, Pflanzenr. (Engler) 4, 105(86): 183. 1924. ≡ *Arabis falcata* A.Rich., Tent. Fl. Abyss. 1: 17. 1847, *nom. illeg.* ≡ ‘*Braya falcata*’ Hochst., Tent. Fl. Abyss. 1: 17. 1847, *nom. inval*. ≡ *Sisymbrium falcatum* E.Fourn., Recherches Anat. Taxon. Fam. Crucifer.: 135. 1865. ≡ *Hesperis falcosa* Kuntze, Revis. Gen. Pl. 2: 934. 1891, *nom. illeg.* ≡ *Stenophragma falcatum* (E.Fourn.) Prantl ex Engl., Abh. Königl. Akad. Wiss. Berlin 1891: 226. 1892.

= *Oreophyton falcatum* f. *depauperatum* O.E.Schulz, Pflanzenr. (Engler) 4, 105(86): 184. 1924. ≡ ‘*Braya falcata* var*. depauperata*’ A.Braun, Pflanzenr. (Engler) 4, 105(86): 184. 1924, *nom. inval.* ≡ *Oreophyton falcatum* var. *depauperatum* (O.E.Schulz) O.E.Schulz, Notizbl. Bot. Gart. Berlin-Dahlem 9: 1107. 1927.

= *Oreophyton falcatum* var. *depauperatum* f. *leiophyllum* O.E.Schulz, Notizbl. Bot. Gart. Berlin-Dahlem 9: 1107. 1927.

  **Distribution.** East Africa (notably Simien and Virunga Mountains, Aberdare Range, Mount Kenya, Mount Elgon, Mount Kilimanjaro).

  **Remark.** Since Kuntze (1891) used the epithet ‘*falcosa*’ instead of ‘*falcata*’, his binomial *Hesperis falcosa* is a replacement name (*nom. nov*.), which becomes simultaneously illegitimate, because the combination *Hesperis falcata* should have been validated instead.

**3. *Oreophyton huetii*** (Boiss.) Guggisberg & Mansion, **comb. nov.** ≡ *Cardamine huetii* Boiss., Diagn. Pl. Orient., ser. 2, 5: 18. 1856. ≡ *Sisymbrium huetii* (Boiss.) Boiss., Fl. Orient. [Boissier] 1: 957. 1867. ≡ *Arabis huetii* (Boiss.) Trautv., Trudy Imp. S.-Peterburgsk. Bot. Sada 2: 496. 1873 . ≡ *Erysimum huetii* (Boiss.) Akinf., Trudy Tiflissk. Bot. Sada 2: 77. 1899. ≡ *Arabidopsis huetii* (Boiss.) N.Busch, Fl. Caucas. Crit. 3(4): 461. 1909. ≡ *Phryne huetii* (Boiss.) O.E.Schulz, Pflanzenr. (Engler) 4, 105(86): 174. 1924. ≡ *Murbeckiella huetii* (Boiss.) Rothm., Bot. Not. 1939: 472. 1939.

= *Arabidopsis pinnatifida* var. *caucasica* Rupr., Mém. Acad. Imp. Sci. Saint Pétersbourg, sér. 7, 15(2) [Fl. Caucasi]: 86. 1869.

= *Arabis araratica* Azn., Magyar Bot. Lapok 17: 2. 1919.

  **Distribution.** Caucasus.

  **Remark.**  *Hutchinsia siliquosa* Bunge (Bot. Erg. Reise Transkaukas. 1: 80. 1857.) is not listed in synonymy of the species, despite its early synonymization with *Oreophyton huetii* by Ruprecht (1869). The protologue by Bunge refers to a single specimen from northwestern Iran (‘*Ssawellan*’ [ = Mount Sabalan]) with few flowers and immature fruits (‘*specimen unicum defloratum fructu immaturo collectum*’) and cannot be certified without seeing the type. The high number of leaf lobes (5–7) reported for the cauline leaves indeed does not fit the description of *O. huetii*.

**4. *Oreophyton omissum*** (B.P.R.Chéron) Guggisberg & Mansion, **comb. nov.** ≡ *Murbeckiella omissa* B.P.R.Chéron, *Murbeckiella* en Ardèche: 14. 2022.

  **Distribution.** Cévennes.

**5. *Oreophyton pinnatifidum*** (Lam.) Guggisberg & Mansion, **comb. nov.** ≡ *Arabis pinnatifida* Lam., Encycl. [J. Lamarck & al.] 1(1): 221. 1783. ≡ *Sisymbrium pinnatifidum* (Lam.) DC., Fl. Franç. [de Candolle & Lamarck], ed. 3, 4(2): 667. 1805, *nom. illeg.* ≡ *Braya pinnatifida* (Lam.) W.D.J.Koch, Syn. Fl. Germ. Helv., ed. 1, 1(1): 50. 1836. ≡ *Descurainia pinnatifida* (Lam.) Webb, Iter Hispan. [Webb]: 75. 1838. ≡ *Arabidopsis pinnatifida* (Lam.) Rupr., Mém. Acad. Imp. Sci. Saint Pétersbourg, sér. 7, 15(2) [Fl. Caucasi]: 86. 1869. ≡ *Hesperis pinnatifida* (Lam.) Kuntze, Revis. Gen. Pl. 2: 935. 1891, *nom. illeg.* ≡ *Stenophragma pinnatifidum* (Lam.) Prantl, Nat. Pflanzenfam. [Engler & Prantl] 3(2): 192. 1891. ≡ *Phryne pinnatifida* (Lam.) Bubani, Fl. Pyren. (Bubani) 3: 177. 1901. ≡ *Murbeckiella pinnatifida* (Lam.) Rothm., Bot. Not. 1939: 469. 1939.

= *Sisymbrium bursifolium* Gouan, Ill. Observ. Bot.: 42. 1773, *nom. illeg*.

= *Sisymbrium dentatum* All., Fl. Pedem. 1: 275. 1785. ≡ *Arabis dentata* (All.) Clairv., Man. Herbor. Suisse: 223. 1811. ≡ *Braya dentata* (All.) Dalla Torre, Anleit. Beob. Alpenpfl.: 62. 1882. ≡ *Arabidopsis dentata* (All.) Dalla Torre, Handb. Atlas Alpenfl.: 115. 1899. ≡ *Erysimum dentatum* (All.) Calest., Nuovo Giorn. Bot. Ital. 15: 379. 1908, *nom. illeg*.

= *Cardamine runcinata* Pourr., Hist. & Mém. Acad. Sci. Toulouse 3: 310. 1788.

= *Sisymbrium bursifolium* Vill., Hist. Pl. Dauphiné 1: 345. 1789, *nom. illeg*.

= *Descurainia peyrusiana* Des Moul., Actes Acad. Roy. Sci. Bordeaux 7(3): 353. 1845. ≡ *Sisymbrium pinnatifidum* var. *peyrusianum* (Des Moul.) E.Fourn., Recherches Anat. Taxon. Fam. Crucifer.: 99. 1865. ≡ *Sisymbrium pinnatifidum* subsp. *lapeyrousianum* (Des Moul.) Rouy & Foucaud, Fl. France [Rouy & Foucaud] 2: 23. 1895, *nom. illeg*. ≡ *Phryne peyrousiana* (Des Moul.) Bubani, Fl. Pyren. (Bubani) 3: 178. 1901, orth. var.≡ *Braya pinnatifida* var*. lapeyrouseana* (Des Moul.) Bonnier, Fl. Compl. France, Suisse & Belg. 1: 70. 1912, *nom. illeg*. ≡ *Phryne pinnatifida* var. *peyrousiana* (Des Moul.) O.E.Schulz, Pflanzenr. (Engler) 4, 105(86): 172. 1924, orth. var..

= *Arabis costae* Willk., Prodr. Fl. Hispan. 3: 820. 1880.

  **Distribution.** Alps, Pyrenees, Massif Central.

  **Remark.** Webb (1838) described *Descurainia pinnatifida* based on material from the Spanish Sierra Nevada, where *Oreophyton pinnatifidum* does not occur; the proposed combination actually refers to *O. boryi*. Des Moulins (1845) narrowed Webb's concept to include only *O. pinnatifidum* in his treatment of *Descurainia*.

  According to BrassiBase, Pourret's (1788) *Cardamine runcinata* is synonymized with *Cardamine raphanifolia*, but according to the corresponding protologues, the former displays simple, dentate leaves (‘*foliis simplicibus, radicalibus* […] *profunde dentatis, caulinis* […] *dentato laciniatis*), as found in *Oreophyton pinnatifidum*, while the latter shows compound leaves (‘*foliis pinnatis*’). Potential original material of *Cardamine runcinata* by Pourret in P-LA and UPS-THUNB indeed corresponds to *O. pinnatifidum*.

  The illegitimate binomials of *Sisymbrium bursifolium* by Gouan (1773) and Villars (1789) were repeatedly confounded with *Sisymbrium bursifolium* L. (1756), which refers to *Sisymbrella dentata*, an annual endemic to Sicily.

  Among the many combinations (homotypic synonyms) derived from *Descurainia peyrusiana* by Des Moulins (1845), those called ‘*peyrousiana*’ can be treated as orthographical errors, the remaining ones (i.e. ‘*lapeyrousianum*’ and ‘*lapeyrouseana*’) being illegitimate. The epithet ‘*peyrusiana*’ refers to the French naturalist Philippe-Isidore Picot de Lapeyrouse, who intensively worked on the flora and fauna of the Pyrenees.

**6. *Oreophyton zanonii*** (Ball) Guggisberg & Mansion, **comb. nov.** ≡ *Erucastrum zanonii* Ball, Bull. Soc. Bot. France 7: 251. 1860. ≡ *Sisymbrium zanonii* (Ball) J.Gay, Bull. Soc. Bot. France 7: 881. 1860. ≡ *Sisymbrium zanonii* (Ball) Caruel, Atti Soc. Ital. Sci. Nat. 5: 149. 1863, *nom. illeg.* ≡ *Hesperis zanonii* (Ball) Kuntze, Revis. Gen. Pl. 2: 935. 1891. ≡ *Sisymbrium pinnatifidum* var. *zanonii* (Ball) Arcang., Comp. Fl. Ital., ed. 2 [Arcangeli]: 264. 1894. ≡ *Erysimum zanonii* (Ball) Calest., Nuovo Giorn. Bot. Ital., ser. 2, 15: 379. 1908. ≡ *Phryne zanonii* (Ball) O.E.Schulz, Pflanzenr. (Engler) 4, 105(86): 173. 1919. ≡ *Murbeckiella zanonii* (Ball) Rothm., Bot. Not. 1939: 471. 1939.

  **Distribution.** Apennines.

Species incertae sedis: *Murbeckiella sousae* Rothm., Bot. Not. 1939: 474. 1939.

## Supplementary Material

mcaf115_Supplementary_Data

## SUPPLEMENTARY DATA

Supplementary data are available at *Annals of Botany* online and consist of the following. Table S1: detailed sample information for newly published data. Table S2: sample information for publicly available data. Figure S1: gene recovery rate. Figure S2: gene selection. Figure S3: polyploid identification. Figure S4: paralogue detection. Figure S5: MULTI2x tree. Figure S6: ITS tree. Figure S7: PLASTID tree. File S1: alignment and partition file in nexus format for plastid sequence data.
