# Supplementary material for: Murbeckiella is dead, long live Oreophyton: origin and systematics of tribe Oreophytoneae (Brassicaceae)
Source: Ann Bot. 2025 Jun 11;138(3):836–50. doi: 10.1093/aob/mcaf115 (PMC13487440; doi:10.1093/aob/mcaf115)
Supplement: Supplementary file 1 [file mcaf115_supplementary_data.zip › supple-figure-capt.docx]

Figure S1: Gene recovery rate - aob-25114-s02.tif

Figure S2: Gene selection - aob-25114-s03.tif

Figure S3: Polyploid identification - aob-25114-s04.tif

Figure S4: Paralog detection - aob-25114-s05.tif

Figures S5: MULTI tree - aob-25114-s06.tif

Figure S6: ITS Tree - aob-25114-s07.tif

Figure S7: PLASTID tree - aob-25114-s08.tif

Table S1: Unpublished data - aob-25114-s09.xlsx

Table S2: Published data - aob-25114-s10.xlsx

File S1: PLASTID alignment - aob-25114-s11.txt
