# Supplementary material for: Murbeckiella is dead, long live Oreophyton: origin and systematics of tribe Oreophytoneae (Brassicaceae)
Source: Ann Bot. 2025 Jun 11;138(3):836–50. doi: 10.1093/aob/mcaf115 (PMC13487440; doi:10.1093/aob/mcaf115)
Supplement: Supplementary file 1 [file mcaf115_supplementary_data.zip › aob-25114-s01.docx]

Prof. Dr. Martin Lysák

AoB Special Issue Editor

*Annals of Botany* journal

Zurich, May 15^th^, 2025

Dear Prof. Lysák

We would like to thank you and the anonymous reviewer for your last suggestions and your positive feedback on our manuscript entitled «***Murbeckiella* is dead, long live *Oreophyton* – Origin and systematics of Tribe Oreophytoneae (Brassicaceae)**» by Nora Walden, Guilhem Mansion, Hektor Schnorf, Marcus A. Koch and Alessia Guggisberg. We have incorporated all the final changes, as requested.

Sincerely yours,

Dr. Alessia Guggisberg, on behalf of all co-authors

Institute of Integrative Biology

ETH Zurich

Universitätstrasse 16

CH-8092 Zürich

Switzerland

[alessia.guggisberg@usys.ethz.ch](mailto:alessia.guggisberg@usys.ethz.ch)

**Referee‘s Review for 25114R:** 

Page 5, line 22: "The combination *Cardamine heterophylla*" - "The name *Cardamine heterophylla*".

Done. 
Page 6, line 1: "Boissier also described *M. huetii* (*Cardamine huetii* Boiss., 1856, p. 18)" - "Boissier (1856, p. 18) also described *Cardamine huetii* Boiss.".

Done. 
Page 26, lines 6-8: "*Murbeckiella* was indeed published much later (Rothmaler, 1939), as a new name for the illegitimate *Phryne* Bubani that Schulz used in his taxonomic treatment" - "*Murbeckiella* was indeed published later (Rothmaler, 1939) to provide a legitimate generic name for the species assigned by Schulz to the illegitimate *Phryne* Bubani".

Done. 
Page 26, line 14: "flowers solitary, forming an ebracteate raceme" - "flowers forming an ebracteate raceme"; solitary means not forming any inflorescence.

Done. 
Page 29, line 22: "combination *Hesperis falcosa* can be seen" - "binomial *Hesperis falcosa* is".

Done. 
Page 29, lines 23-24: "because *Hesperis falcata* should have been used instead" - "because the combination ~~binomial~~ *Hesperis falcata* should have been validated instead"."

Done.
